# Thyroid Function, Prevalent Coronary Heart Disease, and Severity of Coronary Atherosclerosis in Patients Undergoing Coronary Angiography

**DOI:** 10.1155/2015/708272

**Published:** 2015-12-03

**Authors:** Yan Ling, Jingjing Jiang, Minghui Gui, Lin Liu, Qiqige Aleteng, Bingjie Wu, Shanshan Wang, Xiaojing Liu, Xin Gao

**Affiliations:** Department of Endocrinology and Metabolism, Zhongshan Hospital, Fudan University, No. 180 Fenglin Road, Shanghai 200032, China

## Abstract

This study investigated if free T_4_ and TSH concentrations or thyroid function categories were associated with prevalent CHD and the severity of coronary atherosclerosis in a population undergoing coronary angiography. This was a cross-sectional study including 1799 patients who were consecutively admitted and underwent coronary angiography. We evaluated the severity of coronary atherosclerosis using Gensini score. In the entire study population, free T_4_ level was inversely associated with prevalent CHD (OR = 0.95, 95% CI 0.91–0.99, *P* = 0.01) and the natural log-transformed Gensini score (ln(Gensini score)) (*β* = −0.03, 95% CI −0.05–−0.01, *P* = 0.005). The odds of CHD increased gradually across hyperthyroidism, subclinical hypothyroidism, and overt hypothyroidism groups using the euthyroid group as the reference, and the trend is borderline significant (*P* for trend = 0.051). When comparing to the euthyroid group, ln(Gensini score) of the overt hypothyroidism group was significantly higher (*P* = 0.009), but the trend was not significant (*P* for trend = 0.08). A significant association of thyroid function with CHD or ln(Gensini score) in euthyroid patients was not observed. The present study demonstrated an association of thyroid function with prevalent CHD and the severity of coronary atherosclerosis in a population undergoing coronary angiography. However, this association was not observed in euthyroid individuals.

## 1. Introduction

Thyroid hormone has many effects on the cardiovascular system [1]. Thyroid dysfunction results in changes in cardiac contractility, cardiac output, myocardial oxygen consumption, systemic vascular resistance, and blood pressure [1, 2]. The relationship between abnormal thyroid function and coronary heart disease (CHD) has been recognized for a long time, especially in hypothyroidism status due to the associated hypercholesterolemia and hypertension [3, 4]. Even subclinical hypothyroidism [5] and subclinical hyperthyroidism [6] have been related to increased risk of CHD and mortality, although still controversial [7, 8].

Results of some cross-sectional studies of patients undergoing coronary angiography suggested that free thyroxine (T_4_) or free triiodothyronine (T_3_) level was inversely and thyroid stimulating hormone (TSH) concentration was positively associated with the presence of CHD or the severity of coronary atherosclerosis in euthyroid subjects [9–11]. By contrast, one study reported that free T_3_ level was positively associated with the presence and severity of CHD [12]. Another study showed that high level TSH in the reference range was not an independent predictor of CHD [13]. All these studies were conducted in euthyroid subjects with small samples, and the results were conflicting. The HUNT study, a prospective population-based cohort study in Norway, found that low thyroid function within the clinically normal range was associated with increased mortality from CHD in women during 12-year follow-up [14]. However, they found no association of thyroid function with the risk of being hospitalized with myocardial infarction [14]. Therefore, the morbidity finding of the HUNT study does not confirm the suggestion that thyroid function in the normal range is associated with the risk of CHD. More studies are needed to examine the relationship between thyroid function and CHD in euthyroid individuals. From a clinical point of view, the effect of thyroid dysfunction on prevalent CHD may be more important than the effect of the thyroid function in the reference range. To the best of our knowledge, the relationship between different thyroid function, including both normal thyroid function and thyroid dysfunction, and the presence of CHD and the severity of coronary atherosclerosis in a population undergoing coronary angiography is not determined.

Using the data from patients who were consecutively admitted to the Department of Cardiology and underwent coronary angiography, we investigated if free T_4_ and TSH concentrations were associated with prevalent CHD and the severity of coronary atherosclerosis and examined the relationship between thyroid function categories and prevalent CHD and the severity of coronary atherosclerosis in the entire study population and in euthyroid individuals.

## 2. Subjects and Methods

### 2.1. Study Population

Our study enrolled consecutive adults *⩾*30 years of age between March 2013 and November 2013 who underwent coronary angiography for suspected CHD at the Cardiology Department of Zhongshan Hospital in Shanghai, which is affiliated to Fudan University. These patients had chest pain or dyspnea symptoms and were suspected for CHD in primary and secondary hospitals. They were transferred to Zhongshan Hospital for further diagnosis and were first evaluated for CHD at the outpatient department by cardiologists. They underwent routine or dynamic electrocardiogram or coronary computed tomography angiography or exercise treadmill test or stress myocardial perfusion imaging before coronary angiography. If one of these tests was positive, they were hospitalized and had coronary angiography for a definite diagnosis. Data of 2045 patients were collected by a structured interview and medical record review. The exclusion criteria included the following: acute coronary syndrome, using medications (antithyroid medications, thyroid hormone, amiodarone, and glucocorticoid hormone) influencing thyroid function, severe systemic diseases, malignancy, any acute intercurrent illness, and patients with missing data. Finally, 1799 patients were included in the current analysis.

The study was approved by the Ethics Committee of Zhongshan Hospital of Fudan University. The study complied with the Declaration of Helsinki and informed consent was obtained from all patients.

### 2.2. Measurement

#### 2.2.1. Thyroid Function

Free T_4_ and TSH were measured using the electrochemical luminescence method by Modular E170 automatic electrochemiluminescence analyzer (Roche Diagnostics Ltd., Germany). The normal range for TSH is 0.27–4.20 mIU/L and the normal range for free T_4_ is 12–22 pmol/L. Categories of thyroid function were defined as overt hyperthyroidism (TSH < 0.27 mIU/L and free T_4_ > 22 pmol/L), subclinical hyperthyroidism (TSH < 0.27 mIU/L, normal free T_4_), euthyroidism (TSH 0.27–4.20 mIU/L), subclinical hypothyroidism (TSH > 4.2 mIU/L, normal free T_4_), and overt hypothyroidism (TSH > 4.2 mIU/L and free T_4_ < 12 pmol/L).

#### 2.2.2. Coronary Heart Disease

Coronary angiography was performed by using standard Judkins techniques or a radial approach. During cardiac catheterization, nitroglycerine or verapamil was administrated routinely in all cases suspected of having coronary spasm. Angiographic findings were reviewed by two experienced cardiologists who were blinded to the study protocol. Angiography results were divided into CHD (≥50% stenosis in ≥1 coronary artery) group and non-CHD group. We used the Gensini score [15] to assess the severity of stenosis of coronary arteries: it scores it as 1 for 1–25% narrowing, 2 for 26–50%, 4 for 51–75%, 8 for 76–90%, 16 for 91–99%, and 32 for a complete occlusion. This score is then multiplied by a factor, depending on the functional significance of the coronary artery. The multiplying factor for a left main stem lesion is 5. It is 2.5 for proximal left anterior descending artery (LAD) and left circumflex artery (LCX) lesions, 1.5 for a mid-LAD lesion, and 1 for distal LAD, mid/distal LCX, and right coronary artery lesions. The multiplication factor for any other branch is 0.5.

#### 2.2.3. Covariates

Venous blood was drawn in the morning after an overnight fast for at least 12 hours. Fasting glucose, 2-hour postprandial glucose, triglyceride, total cholesterol, and high density lipoprotein cholesterol (HDL-C) were determined by enzymatic methods using Hitachi 7600 biochemistry autoanalyzer (Hitachi High-Technologies Crop., Tokyo, Japan). Low density lipoprotein cholesterol (LDL-C) was calculated according to the Friedewald formula [16]. Glycosylated hemoglobin (HbA1c) was measured using high performance liquid ion exchange chromatography by the Bio-Rad Variant Hemoglobin Testing System (Bio-Rad Laboratories, Hercules, CA).

BMI was calculated as weight (kilograms)/height squared (meter^2^). Systemic arterial hypertension was defined by diagnosis of hypertension made previously by a physician or systolic blood pressure ≥140 mmHg or diastolic blood pressure ≥90 mmHg or treatment with antihypertensive medications. Diabetes mellitus was defined by diagnosis of diabetes made previously by a physician or fasting plasma glucose ≥7 mmol/L or 2-hour postprandial glucose ≥11.1 mmol/L or HbA1c ≥6.5% or use of insulin or oral hypoglycemic agents. A smoking history was defined as current smoking, past smoking, and no smoking ever. Statin use was documented as current use or no use.

### 2.3. Statistical Analysis

Continuous variables were expressed as the mean ± standard error (SE), and categorical variables were expressed as percentages. Comparisons between groups were performed with *t*-test and chi-square test for continuous and categorical variables, respectively. The relationship between continuous T_4_/TSH or thyroid function categories and CHD was determined using logistic regression. The thyroid function categories in the entire population were hyperthyroidism, euthyroidism, subclinical hypothyroidism, and overt hypothyroidism. Subclinical hyperthyroidism and overt hyperthyroidism were combined as one group “hyperthyroidism” due to the small numbers. The euthyroid group was used as the reference. In euthyroid population, the categories of thyroid function were defined as each category representing one-fourth of the width of the reference range of TSH (TSH 0.27–1.27, 1.28–2.28, 2.29–3.29, and 3.30–4.20 mIU/L). The group with TSH between 0.27 and 1.27 mIU/L was used as the reference. Potential confounders were age, sex, BMI, LDL-C, HDL-C, triglyceride, diabetes, hypertension, smoking states, and statin use. The association of continuous T_4_/TSH with the severity of coronary atherosclerosis which was evaluated by Gensini score was assessed by linear regression adjusted for the potential confounders. We used general linear model to determine the association between the categories of thyroid function and the severity of coronary atherosclerosis using the same covariates described above. Gensini score was natural log-transformed before analysis due to the obvious deviation from normal distribution.

## 3. Results

### 3.1. Participants' Characteristics

Characteristics of the study population were presented in Tables 1 and 2. The mean age of the participants was 62.68 ± 0.24 years, and 75.4% were men.

The characteristics of the study population by CHD and non-CHD were presented in Table 1. 82.82% of the participants had coronary angiography confirmed CHD. Patients with CHD were more likely to be male and current and ex-smokers. As expected, patients with CHD were older and had a higher proportion of diabetes and hypertension and a higher level of fasting plasma glucose, 2-hour postprandial plasma glucose, HbA1c, and systolic blood pressure. There were more statin users in the CHD group than non-CHD group. The levels of total cholesterol, LDL-C, and triglyceride were similar between CHD and non-CHD groups, but the HDL-C level was lower in the CHD group. The difference of free T_4_ and TSH levels and thyroid function categories between CHD and non-CHD groups did not achieve statistical significance.

The characteristics of the study population by thyroid function categories were shown in Table 2. Among the study population, 88.33% of participants were euthyroid (*n* = 1589), 0.72% had hyperthyroidism (7 subclinical hyperthyroidism patients and 6 overt hyperthyroidism patients), 9.17% had subclinical hypothyroidism (*n* = 165), and 1.78% had overt hypothyroidism (*n* = 32). Patients with subclinical hypothyroidism and overt hypothyroidism were older than euthyroid patients. Women were more likely to have overt hypothyroidism than men, achieving statistical significance for the comparison between the overt hypothyroidism and euthyroid groups. Patients with overt hypothyroidism had higher Gensini score compared to the euthyroid patients.

### 3.2. Free T_4_, TSH, and Coronary Heart Disease

In the entire population, free T_4_ as a continuous variable was significantly associated with decreased odds of CHD in the multiple logistic regression model, with each one unit increase in free T_4_ predicting a 5% decrease in the odds of CHD (OR = 0.95, 95% CI 0.91–0.99, *P* = 0.01) (Table 3). The association of TSH with CHD was not significant (OR = 1.05, 95% CI 0.99–1.12, *P* = 0.11) (Table 3). FT_4_ was still associated with CHD when FT_4_ and TSH entered into the model together (OR = 0.96, 95% CI 0.92–0.99, *P* = 0.04) (Table 3).

To explore if free T_4_ and TSH in the reference range were associated with CHD, we did analysis in the euthyroid individuals (Table 3). Neither free T_4_ nor TSH was found to be associated with CHD (OR = 0.98, 95% CI 0.92–1.04, *P* = 0.49, and OR = 1.05, 95% CI 0.90–1.23, *P* = 0.51, resp.) (Table 3).

### 3.3. Thyroid Function Categories and Coronary Heart Disease

When comparing to the euthyroid patients, the odds of CHD increased gradually across hyperthyroidism, subclinical hypothyroidism, and overt hypothyroidism groups (OR = 0.74, 95% CI 0.19–2.85; OR = 1.53, 95% CI 0.93–2.52; and OR = 1.59, 95% CI 0.58–4.34, resp.) in the multiple logistic regression model in the entire population, and the trend is borderline significant (*P* for trend = 0.051) (Table 4).

To explore if thyroid function in the reference range was associated with CHD, we did analysis in the euthyroid individuals by grouping them into four categories defined as each category representing one-fourth of the width of the reference range of TSH (Table 4). The thyroid function categories in the reference range were not associated with CHD using the group with TSH between 0.27 and 1.27 mIU/L as the reference (*P* for trend = 0.77).

### 3.4. Free T_4_, TSH, and the Severity of Coronary Atherosclerosis

In the entire population, free T_4_ was inversely associated with ln(Gensini score) in the multivariate linear regression model (*β* = −0.03, 95% CI −0.05–−0.01, *P* = 0.005) (Table 5). TSH was positively associated with ln(Gensini score) in the multivariate linear regression model (*β* = 0.02, 95% CI 0.004–0.04, *P* = 0.02) (Table 5). When free T_4_ and TSH entered into the model together, the association of free T_4_ with ln(Gensini score) was still significant (*β* = −0.02, 95% CI −0.04–−0.004, *P* = 0.02), and the association between TSH and ln(Gensini score) became nonsignificant (*β* = 0.02, 95% CI −0.002–0.03, *P* = 0.08) (Table 5).

We did analysis in euthyroid individuals to investigate if free T_4_ and TSH in the reference range were associated with the Gensini score (Table 5). Neither free T_4_ nor TSH was found to be associated with ln(Gensini score) (*β* = −0.01, 95% CI −0.04–0.02, *P* = 0.36, and *β* = 0.03, 95% CI −0.04–0.10, *P* = 0.36, resp.).

### 3.5. Thyroid Function Categories and the Severity of Coronary Atherosclerosis

**Figure 1:**
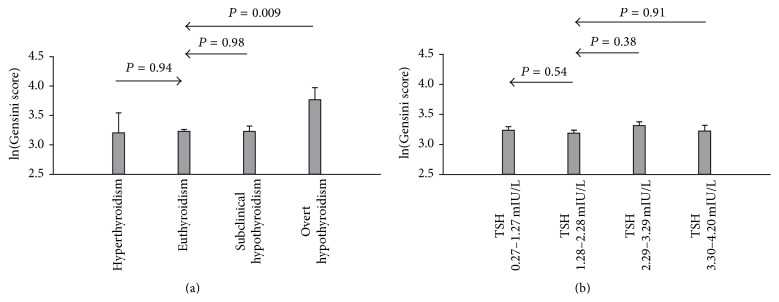
Severity of coronary atherosclerosis by thyroid function categories. (a) Analyses in the entire population. (b) Analyses in euthyroid individuals. Analyses were by general linear model. The dependent variable was the natural log-transformed Gensini score. Values were adjusted mean ± standard error. Multivariate adjustments were made for sex, age, BMI, hypertension, diabetes, low density lipoprotein cholesterol, high density lipoprotein cholesterol, triglyceride, smoking, and statin use. ln(Gensini score): natural log-transformed Gensini score.

When comparing to the euthyroid group, ln(Gensini score) was higher in overt hypothyroidism group (*P* = 0.009) in the general linear model in the entire population (Figure 1), although *P* for trend was not significant (*P* = 0.08). In the euthyroid group, there was no significant difference of ln(Gensini score) between different thyroid function categories (*P* for trend = 0.49) (Figure 1).

## 4. Discussion

In the entire study population, we found that free T_4_ level was inversely associated with prevalent CHD and the severity of coronary atherosclerosis, and there was a significant trend of association of thyroid function categories with prevalent CHD, with lower thyroid function indicating increased risk of CHD. We did not find an association of thyroid function with CHD and the severity of coronary atherosclerosis in euthyroid population.

Thyroid hormone exerts its action on the heart and cardiovascular system through its intranuclear genomic effects and extranuclear nongenomic effects [1, 17]. The ability of thyroid hormone to alter vascular smooth muscle cells and endothelial function are very important [1, 17]. In hypothyroidism, arterial compliance is reduced, which leads to increased systemic vascular resistance and a rise in diastolic blood pressure [3, 17]. Thyroid hormone deficiency is accompanied by a reduced number of low density lipoprotein (LDL) receptors in the liver and a decreased LDL receptor activity, which leads to impaired LDL clearance [17]. As a result, overt hypothyroidism is characterized by hypercholesterolemia and a marked increase in LDL-C [3]. LDL-C is also increased in subclinical hypothyroidism [18]. The lipid profile changes are reversible with thyroid hormone replacement [19, 20]. The dyslipidemia and the diastolic hypertension predispose the hypothyroidism patients to accelerated atherosclerosis and CHD. Although direct evidences about the effect of levothyroxine on CHD are lacking, clinical studies have shown that levothyroxine treatment of subclinical hypothyroidism may have beneficial effects on endothelial function [19] and carotid artery intima-media thickness [21], which are early markers of atherosclerosis.

As discussed above, it is not surprising that free T_4_ level was associated with prevalent CHD and the severity of coronary atherosclerosis in the current study. We also found that TSH level was positively associated with the severity of coronary atherosclerosis, but this association became insignificant after the adjustment of free T_4_. Some studies investigated the direct action of TSH on lipid metabolism, which is closely linked to the development of atherosclerosis and CHD. Tian et al. demonstrated that TSH could upregulate 3-hydroxy-3-methyl-glutaryl coenzyme A reductase in the liver, which indicated a direct role of TSH in the development of hypercholesterolemia [22]. Several studies found that TSH concentration was associated with lipids levels independent of thyroid hormones [23–25]. However, our results do not support the fact that TSH may contribute to the development of atherosclerosis and CHD independent of the function of thyroid hormone. Circulating TSH reflects the negative feedback effects of T_4_ and T_3_ on the pituitary gland and is considered a more sensitive index of thyroid status than free T_4_. However, TSH is a poor measure for estimating the clinical and metabolic severity of primary hypothyroidism [26]. Therefore, it is possible that serum free T_4_ is a more sensitive index of cardiac “thyroid status” than TSH, as shown by the current study and previous studies [19, 27].

Although hypercholesterolemia and increased LDL-C level are one of the important mechanisms underlying the association of hypothyroidism and CHD [3], there was no significant difference of total cholesterol and LDL-C concentrations among different thyroid function categories in the current study. It should be noticed that near 50% of the study subjects use statin treatment in the current study, which may partially explain the similar cholesterol level among different thyroid function categories. We cannot determine whether there were different lipid levels among thyroid function categories before the use of statin or the development of CHD due to the cross-sectional design of the current study. Besides hypercholesterolemia, other mechanisms such as endothelial dysfunction or direct effect on the heart were also very important regarding the relationship between thyroid function and CHD [1].

In the current study, we found that thyroid function in the clinically normal range was not associated with CHD and the severity of coronary atherosclerosis. As a contrary, several previous studies of patients undergoing coronary angiography demonstrated that free T_4_ or free T_3_ level was inversely and TSH concentration was positively associated with the presence of CHD or the severity of coronary atherosclerosis in euthyroid subjects [9–11]. The discrepancy between our study and previous studies can be explained by several aspects. First, the difference may have originated from the heterogeneity of the study subjects regarding age and sex distribution and number and characteristics of the selected subjects. Some studies recruited both stable angina and acute coronary syndrome, and some did not exclude patients with concomitant diseases and medications which can alter thyroid function. Second, the euthyroid population in our study had very high cardiovascular risks. About one-third of the population had diabetes, 70% had hypertension, over 50% used statin, and near 50% were current smokers. As a result, the small effect of variation of thyroid function in the narrow reference range on CHD cannot be captured in the context of multiple classical cardiovascular risks. Third, more prospective cohort studies are needed to determine whether thyroid function in the reference range has an effect on the risk of CHD.

According to the national health and nutrition examination survey, hypothyroidism is a prevalent condition affecting 4.6% of the general population and about 6–8% of the 50–70-year group [28]. The proportion of hypothyroidism was about 11% in the present study population including 9.17% of subclinical hypothyroidism and 1.78% of overt hypothyroidism, which is relatively higher than the prevalence reported in the epidemiological survey. Nonetheless, we cannot recommend routine thyroid function evaluation in individuals undergoing coronary angiography. However, our study suggested that thyroid function screening may facilitate risk stratification in individuals with symptoms of CHD and could provide additional information for selecting the individuals who would benefit from coronary angiography.

The strengths of our study include the large study sample and using coronary angiography to evaluate coronary atherosclerosis. Our study was limited by the cross-sectional design, and a causal relationship cannot be established. In this study, we excluded 29 patients who received levothyroxine therapy and 3 patients who received antithyroid drugs (2 with methimazole and 1 with propylthiouracil) to exclude the effect of medications on thyroid hormone levels. We inferred that the coronary atherosclerosis of these 29 treated hypothyroid patients should be more serious than that of the euthyroid patients. However, there was no significant difference of Gensini score between the levothyroxine treated patients and euthyroid patients. We think that there are two possibilities of explaining this finding. One possibility is that levothyroxine therapy may prevent or slow the development of coronary atherosclerosis and alleviate the degree of coronary lesion. It has been shown by previous study that proinflammatory cytokines decreased and anti-inflammatory cytokines increased after levothyroxine treatment in hypothyroid patients [29]. A decrease in low-grade chronic inflammation may have important clinical relevance due to the known relationship between chronic inflammation, atherosclerosis, and cardiovascular events. Cardiovascular risk factors were also improved after levothyroxine treatment, including glucose, lipids, insulin sensitivity, and soluble intercellular adhesion molecule-1 [30, 31]. More importantly, many studies have shown that levothyroxine replacement in hypothyroid patients improved endothelial functions and reduced arterial stiffness and carotid intima-media thickness [31–34]. These studies provide further evidences that hypothyroidism or lower thyroid hormones may be causally important in the development of CHD.

Another possibility is that hypothyroidism was not serious in the 29 levothyroxine treated patients. The mean body weight for these patients was 70 Kg, but the mean replacement dose was only 60 *μ*g levothyroxine per day and the mean TSH level was 2.59 uIU/mL. So the severity of hypothyroidism in those patients may be close to that of subclinical hypothyroidism. In our study, the Gensini score was not significantly different between the euthyroid group and subclinical hypothyroid group. Similarly, there was no significant difference between euthyroid group and levothyroxine treated group.

We had no data on thyroid autoimmunity, which may have an important effect on the relationship between thyroid function and CHD. It has been shown that thyroid autoimmunity may have some effects on hyperlipidemia and abdominal obesity independent of thyroid function [35]. The association of thyroid peroxidase antibody (TPOAb) and endothelium-dependent arterial dilation in euthyroid Hashimoto's thyroiditis patients also has been reported [36]. Therefore, TPOAb levels should be evaluated in future studies. In addition, we do not know the duration of hypothyroidism of the patients, so the effect of duration of hypothyroidism on coronary atherosclerosis and CHD cannot be examined. Despite the large sample size, the small number of participants with subclinical and overt hyperthyroidism precluded precise estimates for those groups.

In conclusion, the present study demonstrated an association of thyroid function with prevalent CHD and the severity of coronary atherosclerosis in a population undergoing coronary angiography. Our study does not support the fact that thyroid function was associated with prevalent CHD and the severity of coronary atherosclerosis in euthyroid individuals.

## Figures and Tables

**Table 1:** Characteristics of the study population by coronary heart disease and noncoronary heart disease.

Variables	Coronary heart disease	Noncoronary heart disease	*P* value^*∗*^
	(*n* = 1490)	(*n* = 309)	
Male (%)	78.52	60.52	<0.001
Age (years)	62.93 ± 0.26	61.46 ± 0.58	0.02
BMI (Kg/m^2^)	24.79 ± 0.08	24.73 ± 0.19	0.77
Diabetes (%)	36.44	24.92	<0.001
FPG (mmol/L)	5.76 ± 0.04	5.44 ± 0.07	<0.001
2-h PPG (mmol/L)	9.40 ± 0.11	8.34 ± 0.19	<0.001
HbA1c (%)	6.23 ± 0.03	5.98 ± 0.05	<0.001
Hypertension (%)	70.81	63.75	0.02
SBP (mmHg)	129.47 ± 0.36	126.92 ± 0.72	0.003
DBP (mmHg)	77.59 ± 0.23	76.97 ± 0.46	0.25
Total cholesterol (mmol/L)	3.91 ± 0.03	4.01 ± 0.05	0.09
Triglyceride (mmol/L)	1.79 ± 0.04	1.72 ± 0.07	0.40
LDL-C (mmol/L)	2.00 ± 0.02	2.08 ± 0.05	0.12
HDL-C (mmol/L)	1.14 ± 0.01	1.22 ± 0.02	<0.001
Smoking states			<0.001
Nonsmokers (%)	47.41	62.14	
Ex-smokers (%)	8.54	5.18	
Current smokers (%)	44.05	32.69	
Statin use (%)	53.76	35.60	<0.001
Free T_4_ (pmol/L)	15.28 ± 0.06	15.76 ± 0.22	0.11
TSH (uIU/mL)	2.59 ± 0.09	2.38 ± 0.17	0.30
Thyroid function state (%)			0.52
Hyperthyroidism	0.67	0.97	
Euthyroidism	87.92	90.29	
Subclinical hypothyroidism	9.60	7.12	
Overt hypothyroidism	1.81	1.62	

^*∗*^Comparison between coronary heart disease and noncoronary heart disease.

FPG: fasting plasma glucose; 2-h PPG: 2-hour postprandial plasma glucose; HbA1c: glycosylated hemoglobin; SBP: systolic blood pressure; DBP: diastolic blood pressure; LDL-C: low density lipoprotein cholesterol; HDL-C: high density lipoprotein cholesterol; TSH: thyroid stimulating hormone.

**Table 2:** Characteristics of the study population by thyroid function categories.

Variables	Hyperthyroidism	Euthyroidism	Subclinical hypothyroidism	Overt hypothyroidism
	(*n* = 13)	(*n* = 1589)	(*n* = 165)	(*n* = 32)
Male (%)	53.85	76.84	71.52	34.38^*∗*^
Age (years)	60.92 ± 3.05	62.34 ± 0.25	65.42 ± 0.74^*∗*^	66.81 ± 1.94^*∗*^
Free T_4_ (pmol/L)	29.06 ± 5.14^*∗*^	15.44 ± 0.05	14.77 ± 0.14^*∗*^	10.45 ± 0.32^*∗*^
TSH (uIU/mL)	0.06 ± 0.02^*∗*^	1.95 ± 0.02	6.24 ± 0.26^*∗*^	14.39 ± 2.94^*∗*^
Coronary heart disease (%)	76.92	77.34	83.03	84.37
Gensini score	32.27 ± 9.41	37.95 ± 1.09	40.44 ± 3.84	56.61 ± 8.26^*∗*^
BMI (Kg/m^2^)	23.89 ± 0.78	24.78 ± 0.08	24.70 ± 0.24	25.57 ± 0.57
Diabetes (%)	53.85	33.86	36.36	46.88
FPG (mmol/L)	5.66 ± 0.49	5.70 ± 0.04	5.68 ± 0.11	6.13 ± 0.33
2-h PPG (mmol/L)	9.71 ± 1.02	9.20 ± 0.10	9.18 ± 0.30	9.97 ± 0.85
HbA1c (%)	6.46 ± 0.32	6.19 ± 0.03	6.05 ± 0.07	6.47 ± 0.20
Hypertension (%)	84.62	69.23	72.12	68.75
SBP (mmHg)	132.83 ± 3.31	129.04 ± 0.35	128.85 ± 1.03	128.45 ± 2.69
DBP (mmHg)	81.67 ± 2.87	77.48 ± 0.21	77.16 ± 0.70	77.32 ± 1.67
Total cholesterol (mmol/L)	3.39 ± 0.22	3.92 ± 0.02	4.00 ± 0.08	4.23 ± 0.21
Triglyceride (mmol/L)	1.44 ± 0.14	1.79 ± 0.04	1.73 ± 0.11	1.92 ± 0.26
LDL-C (mmol/L)	1.65 ± 0.15	2.00 ± 0.02	2.07 ± 0.06	2.13 ± 0.18
HDL-C (mmol/L)	1.08 ± 0.08	1.15 ± 0.01	1.20 ± 0.03	1.31 ± 0.08
Statin use (%)	53.85	51.23	46.06	43.75
Smoking states				
Nonsmokers (%)	53.85	47.48	66.67	84.38
Ex-smokers (%)	7.69	8.20	6.67	3.13
Current smokers (%)	38.46	44.33	26.67	12.50

^*∗*^ *P* < 0.05 for comparison with euthyroid category.

FPG: fasting plasma glucose; 2-h PPG: 2-hour postprandial plasma glucose; HbA1c: glycosylated hemoglobin; SBP: systolic blood pressure; DBP: diastolic blood pressure; LDL-C: low density lipoprotein cholesterol; HDL-C: high density lipoprotein cholesterol; TSH: thyroid stimulating hormone.

**Table 3:** Odds ratio of prevalent coronary heart disease by free T_4_ and TSH.

Variables	Model 1			Model 2			Model 3		
	OR	95% CI	*P* value	OR	95% CI	*P* value	OR	95% CI	*P* value
Entire population (*n* = 1799)									
Free T_4_	0.95	0.91–0.99	0.01	—	—	—	0.96	0.92–0.99	0.04
TSH	—	—	—	1.05	0.99–1.12	0.11	1.04	0.98–1.10	0.24
Euthyroid population (*n* = 1589)									
Free T_4_	0.98	0.92–1.04	0.49	—	—	—	0.98	0.92–1.05	0.56
TSH	—	—	—	1.05	0.90–1.23	0.51	1.05	0.90–1.23	0.52

Analyses were by multiple logistic regression. Multivariate adjustments were made for sex, age, BMI, hypertension, diabetes, low density lipoprotein cholesterol, high density lipoprotein cholesterol, triglyceride, smoking, and statin use.

TSH: thyroid stimulating hormone.

**Table 4:** Odds ratio of prevalent coronary heart disease by thyroid function categories.

Entire population				Euthyroid population			
Thyroid function categories	*n*	OR	95% CI	Thyroid function categories	*n*	OR	95% CI
Hyperthyroidism	13	0.74	0.19–2.85	TSH 0.27–1.27 mIU/L	422	1	Reference
Euthyroidism	1589	1	Reference	TSH 1.28–2.28 mIU/L	625	1.039	0.74–1.47
Subclinical hypothyroidism	165	1.53	0.93–2.52	TSH 2.29–3.29 mIU/L	388	0.85	0.59–1.24
Overt hypothyroidism	32	1.59	0.58–4.34	TSH 3.30–4.20 mIU/L	154	1.40	0.81–2.40
*P*_trend_	0.051			*P*_trend_	0.77		

Analyses were by multiple logistic regression. Multivariate adjustments were made for sex, age, BMI, hypertension, diabetes, low density lipoprotein cholesterol, high density lipoprotein cholesterol, triglyceride, smoking, and statin use.

**Table 5:** Correlates of free T_4_ and TSH to the severity of coronary atherosclerosis.

Variables	Model 1			Model 2			Model 3		
	*β*	95% CI	*P* value	*β*	95% CI	*P* value	*β*	95% CI	*P* value
Entire population (*n* = 1799)									
Free T_4_	−0.03	−0.05–−0.01	0.005	—	—	—	−0.02	−0.04–−0.004	0.02
TSH	—	—	—	0.02	0.004–0.04	0.02	0.02	−0.002–0.03	0.08
Euthyroid population (*n* = 1589)									
Free T_4_	−0.01	−0.04–0.02	0.36	—	—	—	−0.01	−0.04–0.02	0.42
TSH	—	—	—	0.03	−0.04–0.10	0.36	0.03	−0.04–0.10	0.43

Analyses were by multiple linear regression. The dependent variable was the natural log-transformed Gensini score. Multivariate adjustments were made for sex, age, BMI, hypertension, diabetes, low density lipoprotein cholesterol, high density lipoprotein cholesterol, triglyceride, smoking, and statin use.

TSH: thyroid stimulating hormone.
